# Stem Cell-Derived Organoids

**DOI:** 10.1155/2024/9798375

**Published:** 2024-01-21

**Authors:** Yujing Li, Xuekun Li, Yunhee Kang, Shu Jiang

**Affiliations:** ^1^Department of Human Genetics, Emory University School of Medicine, 615 Michael Street, Atlanta, GA 30047, USA; ^2^The Institute of Translational Medicine and The Children's Hospital, School of Medicine, Zhejiang University, Kaixuan Road 268, Hangzhou, Zhejiang 310029, China; ^3^1089 Chaguang Road, Nanshan District, Shenzhen City 518055, Guangdong Province, China

Conventionally, 2D-cultured cells and animal models, such as Drosophila, Zebrafish, and mice, have played a vital role in understanding the developmental and pathophysiological mechanisms underlying human diseases. However, the shortcomings of these models, including the lack of spatial information and genetic/physiological disparities between animals and humans, hinder the translation of research findings into clinical applications, particularly for complex and chronic diseases. This disparity becomes apparent in the underwhelming results of clinical trials, specifically those investigating mGluR5 antagonists for fragile X syndrome, which were based on insights gained from animal model research.

To overcome the limitations mentioned above, organoids have emerged as a promising alternative. Organoids are miniature and simplified versions of organs generated *in vitro*, faithfully recapitulating the microanatomy of their *in vivo* counterparts. By starting from human embryonic stem cells or human induced pluripotent stem cells, researchers can develop organoids that closely mimic human organ development, providing invaluable platforms for biomedical studies, investigations into genetic disorders and diseases, drug screening and development, and regenerative medicine.

Thus far, various types of organoids have been successfully generated, representing a wide range of organs, including the brain, lung, intestine, stomach, liver, pancreas, kidney, and more. Despite the initial advancements in 3D culture systems, there are still existing engineering and conceptual challenges that hinder the efficiency and quality of organoids in accurately mimicking human organ development—an essential aspect for therapeutic purposes. Key limitations in current *in vitro* culture systems include the lack of vascularization, cellular diversity, tissue maturity, and overall functionality.

Recent advancements have brought about exciting developments in the establishment of functional vascular-like networks within organoids. These breakthroughs have been achieved through various approaches, including the addition of vascular endothelial growth factor and wingless-related integration site 7a (Wnt7a) to the culture media, coculturing with endothelial cells (ECs), culturing organoids on ECs beds, transplanting them into vascularized animal tissues, or employing genetic engineering techniques such as inducing the expression of human ETS variant 2. The vascularized organoids could more accurately mimic features of their counterparts *in vivo*, exhibiting better organization, reduced hypoxia and apoptosis. This breakthrough sheds light on the generation of organoids with higher efficiency and greater resemblance to human organogenesis.

These breakthroughs have served as a catalyst for scientists to redirect their attention from animal models to human-centric models, with the objective of studying pathological mechanisms in the context of human genetic disorders and diseases with greater precision. As a result, it is of paramount importance to initiate a special issue that specifically highlights the advancements made in the generation and utilization of organoids derived from stem cells.

In this special issue, a total of 10 articles were published, including four original research and six review articles covering topics on the generation techniques of human organoids and their diverse applications in biomedical studies.

The intestine plays crucial roles in the digestive system and is highly prone to various diseases caused by enteric pathogens. However, studying the pathophysiology of these pathogens within host cells using animal models has been extremely challenging. Fortunately, the emergence of human intestinal organoids (HIOs) holds great promise as they faithfully mimic key features of the human intestinal mucosa. This collection comprises four articles that specifically center around HIOs. It includes two research articles authored by Bruegge et al. and Kandilogiannakis et al., as well as two review articles by Hentschel et al. and Hisa et al. Bruegge et al.'s article investigated bacterial stimulation experiments and revealed that cryopreserved HIOs were not suitable replacements for animal models. This was attributed to their premature properties and heightened proinflammatory response to bacterial infection. On the other hand, Kandilogiannakis et al. determined that early passages of HIOs provided optimal opportunities to study inflammatory and fibrotic responses during HIO development. Regarding the review articles, Hentschel et al. provided a comprehensive update on HIO-based tissue engineering. This included insights into the latest pathophysiological characteristics observed in infected HIOs, as well as an exploration of the limitations associated with this innovative *in vitro* model. Hsia et al., on the other hand, focused on the advancements made in the transplantation of HIOs and various other organoids, considering the limitations posed by the restricted available space.

Exosomes, as vital components of the paracrine pathway, play a pivotal role in intercellular communication and the transfer of genetic material. Their involvement in bone injury repair is significant, and their relevance in tissue engineering has been increasingly acknowledged.

Shao et al. found that exosomes derived from mesenchymal stem cells (MSCs) obtained from the infrapatellar fat pad enhance the proliferation of rabbit chondrocytes. Interestingly, exosomes derived from MSCs that were pretreated with KGN (wingless-related integration site 7a (Wnt7a)) exhibited an even more remarkable stimulation of proliferation. Moreover, the combined treatments demonstrated significant potential in facilitating the repair of articular cartilage damage, thus illuminating a promising strategy for future therapeutic applications.

Zhang et al. offer an up-to-date and comprehensive overview of the methodological aspects involved in the generation of adipose-derived stem cells (ADSCs). Additionally, it emphasizes significant advancements, perspectives, and challenges pertaining to the preclinical and clinical applications of ADSCs in the field of regenerative medicine.

Zhao et al. highlight the advances in salivary gland organoids, discussing both the advancements and limitations associated with their development. The authors address the promising potential of salivary gland organoids in the field of regenerative medicine.

In addition, two independent review articles by Ma et al. and Xu et al. focus on the recent progress made in the generation methodology and applications of human brain organoids. These articles cover a wide range of topics, including basic biological studies, disease modeling, and high-throughput drug screening using brain organoids. These reviews provide valuable insights into the current state of research and the potential of human brain organoids in various areas of study.

As the articles in this issue highlight, human organoids offer fundamental advantages over *in vitro* cells and animal models in biomedical research, and they hold immense promise for potential clinical therapy and regenerative medicine. Continued development and innovation in the generation of human organoids will enable them to replicate the features of their *in vivo* counterparts more accurately. By ensuring high quality, organoids can significantly contribute to the understanding of the physiopathology underlying human diseases, enhance the efficiency of drug screening, advance regenerative medicine, and provide a novel avenue for clinical therapy.

The authors of this special issue hold a strong desire that the research and review articles published here will provide valuable and convenient information for readers interested in this closely related research field, both technically and scientifically.

*Yujing Li* *Xuekun Li* *Yunhee Kang* *Shu Jiang*

